# Investigation of Raman Spectroscopy (with Fiber Optic Probe) and Chemometric Data Analysis for the Determination of Mineral Content in Aqueous Infant Formula

**DOI:** 10.3390/foods9080968

**Published:** 2020-07-22

**Authors:** Ming Zhao, Saif Shaikh, Renxi Kang, Maria Markiewicz-Keszycka

**Affiliations:** 1 Department of Food Chemistry and Technology, Teagasc Food Research Centre, Ashtown, Dublin 15, Ireland; 2 School of Biosystems and Food Engineering, University College Dublin, Belfield, Dublin 4, Ireland; saif.shaikh@ucdconnect.ie (S.S.); renxi.kang@ucdconnect.ie (R.K.); 3 School of Agriculture & Food Science, University College Dublin, Belfield, Dublin 4, Ireland; maria.markiewicz-keszycka1@ucd.ie

**Keywords:** Raman spectroscopy, chemometrics, infant formula, minerals, ICP-AES analysis

## Abstract

This study investigated the use of Raman spectroscopy (RS) and chemometrics for the determination of eight mineral elements (i.e., Ca, Mg, K, Na, Cu, Mn, Fe, and Zn) in aqueous infant formula (INF). The samples were prepared using infant formula powder reconstituted to concentrations of 3%–13% *w/w* (powder: water) (*n* = 83). Raman spectral data acquisition was carried out using a non-contact fiber optic probe on the surface of aqueous samples in 50–3398 cm^−1^. ICP-AES was used as a reference method for the determination of the mineral contents in aqueous INF samples. Results showed that the best performing partial least squares regression (PLSR) models developed for the prediction of minerals using all samples for calibration achieved R^2^CV values of 0.51–0.95 with RMSECVs of 0.13–2.96 ppm. The PLSR models developed and validated using separate calibration (*n* = 42) and validation (*n* = 41) samples achieved R^2^CVs of 0.93, 0.94, 0.91, 0.90, 0.97, and 0.94, R^2^Ps of 0.75, 0.77, 0.31, 0.60, 0.84, and 0.80 with RMSEPs of 3.17, 0.29, 3.45, 1.51, 0.30, and 0.25 ppm for the prediction of Ca, Mg, K, Na, Fe, and Zn respectively. This study demonstrated that RS equipped with a non-contact fiber optic probe and combined with chemometrics has the potential for timely quantification of the mineral content of aqueous INF during manufacturing.

## 1. Introduction

Infant formula (INF) is intended for infants and young children and can be a complete or partial substitute for human milk [1]. Dairy-based INF consists of almost all macronutrients (i.e., fats, carbohydrates, and proteins) and micronutrients (i.e., vitamins and minerals) [2]. To simulate human milk closely, commercial infant formulas are fortified with essential micronutrients (including trace minerals) and have the content of some macro minerals (e.g., Ca, Na, etc.) reduced to satisfy nutritional requirements [3]. Minerals play a vital role in forming essential parts of many enzymes and biological molecules of the human metabolic system for body tissue growth and other physiological functions. Therefore, the quantification of INF elemental composition is critical for quality control during manufacture. 

Multi-element determinations at the ultra-trace level can be achieved using atomic absorption spectroscopy (AAS), inductively coupled plasma-atomic emission spectroscopy (ICP-AES) and ICP-mass spectroscopy (ICP-MS). These techniques have been well investigated for the quantification of trace elements in milk and INF [4,5,6,7,8,9,10]; however, they are only suitable for laboratory use as they are expensive, time-consuming and require sophisticated instrumentation and procedures; thus, they cannot meet the requirements for timely measurement during manufacturing.

In the dairy industry, powdered INF is usually manufactured using either a dry blending process or a wet mixing-spray drying process [11]. The wet mixing-spray drying process currently remains the most widely used method of powdered infant formula production [11]. From a quality control perspective, one of the critical disadvantages of the dry blending process is that the macro- and micro-nutrients may segregate in varied layers of milk powder during transportation and storage due to their different densities resulting in an inhomogeneous product for consumers [12]. An advantage of the wet mixing-spray drying method is that all the production processes, including wet mixing, concentration by evaporation, and spray drying, can be effectively controlled to attain homogenized and stable final products [12]. Non-destructive process analytical techniques are required to monitor nutrient levels at critical points during manufacture to ensure that the product produced is within specification.

Previously, process analytical methods have been widely investigated to verify nutrient levels of milk and INF. Near-infrared spectroscopy (NIR) has been successfully applied to predict various constituents in milk, including fat (R^2^CV—0.99), protein (R^2^CVs—0.98–0.99), albumin, blood urea nitrogen (R^2^CV—0.82), lactose (R^2^CVs—0.86–0.92), glucose, and somatic cell count (R^2^CV—0.85) [13,14,15]. Mid-infrared (MIR) spectroscopy has been employed for milk compositional analysis with high accuracy in the prediction of specific fatty acids (R^2^CV—0.29–0.97) and poor accuracy on the content of major minerals (R^2^CV—0.41–0.48) [16]. Other studies using MIR reported moderate accuracy for the prediction of macroelements in bovine milk (R^2^CV—0.42–0.71) [17] and high accuracy for the prediction of milk protein composition (R^2^CV—0.59–1) [18]. Raman spectroscopy (RS) has also been reported for prediction of nutritional parameters (fat, protein, energetic values, and carbohydrates) in INF and milk powder [19,20,21,22], milk fat or whey in liquid milk [23] and melamine adulterants in liquid and powdered milk [24,25,26]. A few publications have studied the use of RS to detect mineral contents in dairy products. A study by Smith et al. (2013) has been reported using Fourier-transformed RS to detect and quantify calcite in milk powder [27]. Laser-induced breakdown spectroscopy (LIBS) has also been reported to quantify selected minerals in INF powder and INF premixes [28,29] and yielded an R^2^CV value of 0.90 with RMSECV of 680 mg/kg (ppm) for the prediction of Ca; R^2^CVs of 0.83 with RMSECVs of 16.1 mg/kg (ppm) and 226.4 mg/kg (ppm) for the prediction of Cu and Fe, respectively. To date, almost all the published studies using process analytical technology for quantification of mineral elements in infant formula were carried out on dry powder samples. To the authors’ knowledge, no information is currently available for quantifying mineral contents of aqueous INF using RS.

As a non-invasive technique, RS has considerable advantages for the analysis of aqueous samples compared to other methods because water has weak Raman scattering properties. Unlike infrared spectroscopy, this technique is not limited by water interference. For trace element determination, RS can provide sophisticated spectral information of chemical composition with high resolution [30]. It has also been used to determine minerals on planetary surfaces [31,32], in bones, [33] and oil paintings [34]. Recently, RS combined with chemometrics has been reported to determine Ca content in chicken bone and meat mixtures (R^2^CV of 0.775 with RMSECV of 0.33%) [35] and to predict Ca content in powdered infant formula accurately (R^2^CV of 0.954 with RMSECV of 0.490 mg/g) [36]. Therefore, RS has the potential to determine mineral contents in aqueous INF during wet-mixing before the drying process for real-time quality control. The objective of this study is to investigate the potential of RS with chemometrics for the determination of mineral contents in aqueous INF samples.

## 2. Materials and Methods 

### 2.1. Sample Preparation

Infant formula powder (INFP) samples (*n* = 14) were produced using a pilot-scale research facility at Teagasc food research center (Moorepark, Cork) and stored in sealed bags at ~15 °C for three months. Moreover, five commercial INFPs (i.e., Aptamil, SMA, Cow&Gate, Mamia, and Similac) were purchased from local supermarkets in Ireland. Altogether, 19 INFP samples were included in this study. The moisture content of each powder was measured using a halogen rapid moisture analyzer (HR-83 Halogen, Mettler Toledo, Switzerland) at 105 °C until a constant weight (<1 mg) was attained in 140 s. For aqueous sample preparation, powder samples were weighed and dissolved in 200 mL of deionized water, considering moisture content to obtain solutions at five concentrations, i.e., 3%, 5%, 8%, 10%, and 13% *w/w*. During homogenization, an overhead 4-blade impeller of a high shear mixer (Silverson L4R, Silverson Machine Ltd., Bucks, UK) was positioned at the center of the deionized water and operated at 400 rpm; the weighed powder was added and mixed at ambient temperature (∼15 °C) for 20 min. In total, eighty-three aqueous samples were prepared for this study including 75 aqueous samples prepared from 15 powder samples (including 14 produced INFP samples and one of the commercial INFP samples) at concentrations of 3%, 5%, 8%, 10% and 13% *w/w*; 7 aqueous samples prepared from 7 powder samples (including five commercial INFP and two of the produced 14 INFP) at a concentration of 13 % *w/w*, and one aqueous sample prepared from one powder sample at a concentration of 8% *w/w* due to the limited amount of these particular INFP samples. After sample preparation, all aqueous samples were kept overnight at 4 °C before measurements.

### 2.2. Raman Spectral Data Acquisition of Aqueous INF Samples

Raman spectral data were collected using a non-contact fiber optic probe on the surface of aqueous INF samples. A fiber optic probe and the connected coaxial cable were fixed on a probe stand to avoid effects caused by subtle changes of instrument configuration. Each sample (10 mL) was transferred into an aluminum cylindrical container (45 mm diameter, 50 mL volume) and placed on a variable height platform under the probe. Samples were raised so that their surface was 2 mm away from the quartz screen of the probe head. A DXR SmartRaman spectrometer (ThermoFisher Scientific UK Ltd., Loughborough, UK) was used for spectral data acquisition. The system was equipped with a diode laser operating at 780 nm and a charge-coupled device (CCD) detector. The sample surface was exposed under the aperture (50 μm slit) of the fiber optical probe accessory. For each scan, a 120 mW laser power was used; spectra of each sample were automatically accumulated until the maximum signal to noise ratio of the averaged spectrum was obtained. All aqueous samples were scanned in random order at ca. 20 °C. Raman intensity counts per second (cps) were recorded in the wavelength range of 50−3380 cm^−1^ at 2 cm^−1^ increments. Automatic cosmic spikes removal, instrument control, spectral acquisition, and file conversion were operated using OMNIC software (v 9.2.98; Thermo Fisher Scientific Inc., Madison, WI, USA). Each sample was scanned twice at different locations of the aqueous surface. The averaged spectrum of the duplicate was used in the subsequent chemometric analysis.

### 2.3. Inductively Coupled Plasma Atomic Emission Spectroscopy (ICP-AES) Analysis

#### 2.3.1. Preparation of Reagents and Standard Solutions for ICP-AES

Deionized water (resistivity−18.2 MΩ-cm) was produced using a Barnstead™ Smart2Pure™ water purification system (Thermo Scientific, Waltham, MA, USA) for the preparation of reagents and standard solutions for ICP-AES analysis. To avoid mineral contamination, all glassware sets used in this study were washed using 2% *v/v* detergent (RBS 25 Concentrate, CHEMICAL PRODUCTS R. Borghgraef S.A., Brussels, Belgium) diluted with tap water followed by rinsing, air-drying and soaking in 10% *v/v* HNO_3_ prepared from 65% *v/v* HNO_3_ (code: N/2185/PB17, Fisher Scientific, Loughborough, Leicestershire, UK). Before use, glassware was rinsed three times with deionized water and dried in a thermal cabinet. For ICP-AES instrumental calibration, standard solutions were prepared using the ICP multi-element standard stock solution of 1000 ppm (Certipur^®^, Merck KGaA, Darmstadt, Germany) diluted to 1.25, 2.5, 5, 12.5, 25 and 50 ppm respectively using 5% *v/v* HNO_3_ prepared from 69% *v/v* HNO_3_ (code: N/2320/PB17, Fisher Scientific, Loughborough, Leicestershire, UK). Standard solutions were stored overnight in ash-free polypropylene containers at ~4 °C before ICP-AES measurements. 

#### 2.3.2. Dry Digestion 

Each aqueous INF sample (10 mL) was transferred into a porcelain crucible (25 mL) and heated in a chamber furnace (Temperature Controller type 201, Carbolite, UK) at 650 °C for three hours until white ashes were obtained.

Ashed samples were digested using 5% *v/v* HNO_3_. The acid digestion solution of each sample was prepared by dissolving ash residue in 25 mL of 5% *v/v* HNO_3_ for analysis of INFP minerals at low concentrations (<140 ppm), e.g., Mg, Cu, Fe, Zn, Mn, etc. [37]. For analyzing minerals contained in INFP at higher concentrations (>350 ppm), e.g., Ca, Na, K, etc., the same acid digestion solution was diluted 1:10 *v/v* using 5% *v/v* HNO_3_. All solutions were kept in the polypropylene containers at 4 °C before ICP-AES analysis.

#### 2.3.3. ICP-AES Apparatus and Working Conditions 

A Vista RL (radially viewed plasma) CCD simultaneous ICP-AES (Varian Inc., Mulgrave, Victoria, Australia) spectrometer powered by a 40 MHz radiofrequency generator at 1.2 kW was used for the determination of all minerals. This equipment employed an echelle polychromator with a cross dispersion prism to form a two-dimensional diffraction pattern across its focal plane. A charge-coupled device detector was equipped to detect electromagnetic signals at a wavelength range of 167–784 nm. Argon gas was used as a plasma source at a flow rate of 15 L/min, and the auxiliary gas flow rate was 1.5 L/min. Before the operation, instrumental calibration was carried out to develop standard curves with < 10% of maximum error and >0.995 correlation coefficient values. During the analysis, the prepared standard solution or acid digestion solution was introduced into the system via a SeaSpray concentric nebulizer (Glass Expansion, Pocasset, MA, USA) operated with argon aerosol gas (200 kPa) at a 0.75 L/min flow rate. Sample aspiration was forced using an Alitea v-10R peristaltic pump at 15 rpm with a 1.4 mL/min sample delivery rate. To avoid nebulizer contamination and clogging, a sample uptake delay (15 s) and rinse time (10 s) with deionized water were applied between each measurement. The signals were obtained at an observation height of 13 mm above the load coil and were corrected using the embedded background correction method. Calibration curves were developed using a blank solution (5% *v/v* HNO_3_) and multi-element standards of 12.5, 25, and 50 ppm for elements present at higher concentrations and the blank and multi-element standards of 1.25, 2.5, and 5 ppm for the elements at lower concentrations. For sample analysis, the optimal wavelength for monitoring each mineral element was selected from three relevant wavelengths suggested by the supplied software of the ICP-AES system. The optimal wavelength for each specific element was decided based on the highest linearity (R^2^~1) of the developed calibration curves. Each sample was measured in triplicate; the mean value of each mineral element was acquired, and the repeatability value for each element detection was also calculated [38]. The ICP-AES instrument operations and data acquisition were performed using ICP-Expert version 4.1.0 software (Varian, Inc., Australia). Data were exported as .csv files and imported into Matlab 2018a (The Mathworks, Natick, MA, USA) for both statistic and chemometric analysis.

### 2.4. Accuracy Determination on ICP-AES Analysis

Accuracy of the ICP-AES analysis method was investigated by analyzing a certified reference material (skim milk powder ERM-BD 150, European Commission, JRC, IRMM, Retieseweg, Geel, Belgium). After dry matter analysis, the certified reference material (1 g) was transferred into a porcelain crucible (100 mL) and heated at 400 °C for 20 min using a hot plate (MaXtir 500H, Daihan scientific, Gangwon-do, KOREA) until no further smoke was observed. Then the crucible was placed into a furnace (Temperature Controller type 201, Carbolite, UK) to heat the material at 650 °C for 3.5 h until white ashes were obtained. For ICP-AES analysis, dry digestion and ICP-AES measurements were carried out following the procedures described in Section 2.3. The analysis was carried out in triplicate; the mean value of each mineral element was acquired and compared to the certified reference. Accuracy of ICP-AES analysis was defined by percent recovery (%R) [9,39], which was described as: %R = (measured value of a mineral/certified value of a mineral) *100%.

### 2.5. Control Experiments on Chloride Salts Using RS

1M chloride salt solutions were prepared using CaCl_2_, MgCl_2_, KCl, NaCl, MnCl_2_, iron(III)chloride hexahydrate (FeCl_3_ + 6 H_2_O), CuCl_2_, and ZnCl_2_ crystals (purity ≥ 98.0%; Sigma-Aldrich, 3050 Spruce St., St. Louis, MO, USA) dissolved in deionized water. These chloride salt solutions were separately kept in polypropylene containers. Before Raman measurements, each solution was well shaken using a vortex mixer (VWR International Ltd., Blanchardstown, Dublin, Ireland) for 1 min to ensure complete dissolution. During the experiment, 10 mL of each solution was transferred into a 50 mL quartz beaker with aluminum foil smoothly covering the outer surface and then placed on an elevating frame under the fiber optic probe stand of the Raman spectrometer (ThermoFisher Scientific UK Ltd., Loughborough, UK). The sample was raised to ensure that its surface was 2 mm away from the quartz screen of the probe head. Detailed measurement procedures and instrumental settings are described in Section 2.2. Each sample was scanned twice at two different surface locations; the mean of these duplicate spectra was used for chemometric analysis.

### 2.6. Chemometric Analysis

Raw Raman spectra of all the measurements were imported into Matlab 2015a (The Mathworks, Natick, MA, USA). The mean spectrum of each sample was calculated. Baseline correction on raw data was carried out using asymmetric least squares correction (AsLs), adaptive iteratively reweighted penalized least squares (airPLS) [40], Savitzky–Golay (S.G.) first derivatives calculated using a 2nd-degree polynomial with 7 smoothing points, and baseline offset correction (BOC). For the exploration of the potential of Raman spectroscopy for mineral prediction, PLSR models were developed using the nonlinear iterative partial least squares (NIPALS) algorithm. Models were developed using pre-processed Raman spectral data (X variables) of 50–3398 cm^−1^ and 50–1800 cm^−1^ frequency ranges based on observed spectral signal intensities. The ICP-AES measured values of each mineral element for all INF aqueous samples (*n* = 83) were used as individual Y variable. For further investigations, PLSR models were also developed using calibration samples (*n* = 42) and validated using the rest samples (*n* = 41); quasi-random selection was used to split the whole sample group into calibration and validation sample sets. Venetian blind cross-validation was performed to evaluate the performance of PLSR models. Parameters such as root mean square error of calibration (RMSEC) and cross-validation (RMSECV), the coefficient of determination on calibration (R^2^C), cross-validation (R^2^CV) and prediction (R^2^P) were calculated. The bias of cross-validation and prediction was also determined. The satisfactory prediction results from a PLSR model were expected to have R^2^ values close to 1, RMSE values, and bias close to 0. Robust PLSR models were developed using a small number of latent variables (PLS loadings) [41]. Improvements in the performance of PLSR models were attempted using a reduced number of Raman spectral variables which were selected by variable importance on projection (VIP) [42], significance multivariate correlation (sMC), and Martens’ uncertainty test [43]. 

Chemometrics was also employed for the specification of mineral-related Raman spectral variables. Based on PLSR modeling, the Raman spectral frequencies of significant regression coefficient intensities for each mineral element prediction were used to compare with the pre-processed Raman spectral signals of the chloride salt solution (contained with the mineral ions) in order to verify their common locations of Raman spectral signals. 

## 3. Results and Discussion

### 3.1. Raman Spectra of Aqueous INF Samples

**Figure 1:**
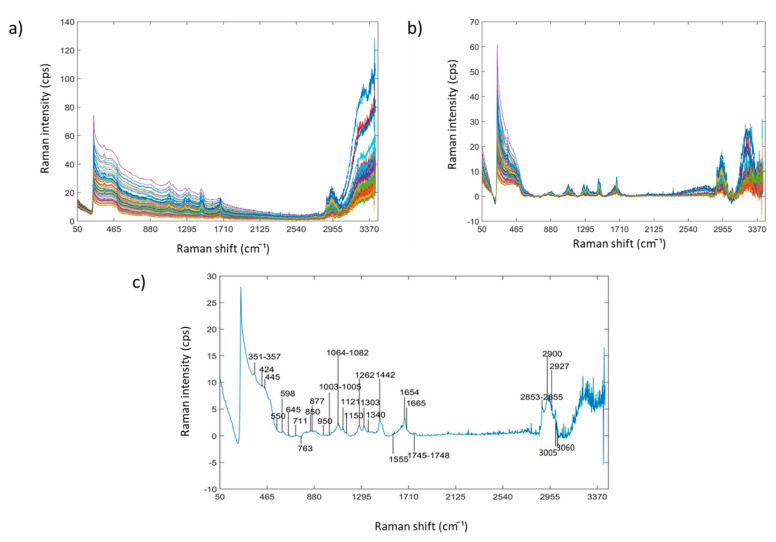
(**a**) Raw Raman spectra in 50–3398 cm^−1^ of all aqueous INF samples (*n* = 83); (**b**) Raman spectra in 50–3398 cm^−1^ pre-treated by AsLs baseline correction; (**c**) Averaged Raman spectrum in 50–3398 cm^−1^ pre-treated by AsLs baseline correction.

Raw Raman spectra (50–3398 cm^−1^) of aqueous INF samples (*n* = 83) are shown in Figure 1a. Fluorescence and multiplicative effects were removed using AsLs baseline correction (Figure 1b). The mean spectrum for all samples in 50–3398 cm^−1^ region is shown in Figure 1c. Raman spectral bands at 351–357, 445, 850, 877, 950, and 1064–1082 cm^−1^ are assigned to the vibrational mode of the glycosidic bond of α or β lactose [44,45]. Raman bands at 445 and 598 cm^−1^ may also be related to δ (C-C-C) and τ (C-O) bonds [46]. 645, 763 and 877 cm^−1^ are also relevant to δ (C-C-O) or δ (C-C-H) bonds of tryptophan [21,46]; 950 cm^−1^ has been assigned to δ (C-O-C), δ (C-O-H) and ν (C-O) bonds; 1065–1082 and 1121cm^−1^ has also been assigned to δ (C-O-H), ν (C-O) and ν (C-C) of aspartic and glutamic acid [47]. Raman bands at 1003–1005 cm^−1^ have been strongly related to the ring-breathing structure of phenylalanine reported in many previous studies [21,41,47,48]. Peaks at 1262, 1303, 1442 and 1745–1748 cm^−1^ are assigned to γ (CH_2_), τ (CH_2_), δ (CH_2_) and ν (C=O) bonds of aliphatic chains in lipids and amino acid residues, respectively [21,48]. Raman bands at 1555 and 1654−1665 cm^−1^ are related to δ (N-H) and ν (C=N) of Amide II, and ν (C=O) of Amide I, respectively [21,47]. The regions of 2853–2855 cm^−1^, 2900–2927 cm^−1^, and around 3005 cm^−1^ may be attributed to symmetric ν (CH_2_), asymmetric ν (CH_3_), and the symmetric γ (CH_2_) vibrational mode of aliphatic chain and aromatic structures of lipids [23]. The Raman band at 3060 cm^−1^ has been assigned to ν (N-H) of amino groups [20]. 

### 3.2. Results of ICP-AES Analysis

Statistics of the ICP-AES results on the eight selected mineral elements determined from all the aqueous INF samples (*n* = 83) are summarized in Table 1. For the determination of each element, the associated optimal wavelengths for monitoring individual elements are also listed in Table 1. The determination limits for each element over all samples are described as Min ± repeatability and Max ± repeatability. The determined concentration ranges of Ca (3.002–25.295 ppm), Na (0.144–9.133 ppm) and K (0.129–17.319 ppm) are within designed ICP-AES multi-elements standard higher concentration calibration range (0- 50 ppm) of the mineral contents. The concentration ranges of Mg (0.281–2.537 ppm), Fe (0.016–3.13 ppm), Zn (0.045–2.575 ppm), and Cu (0.009–0.563 ppm) are within the designed lower concentration ICP-AES calibration range (0–5 ppm). Mn could not be determined due to the detection limit of the ICP-AES method used in this work. Therefore, except Mn, the reference values of the other seven elements were used in further PLSR modeling.

### 3.3. Accuracy of ICP-AES Analysis

The accuracy of ICP-AES analysis in the current work was evaluated by comparing the element determination of a certified material (skim milk powder, ERM-BD 150, European Commission, JRC, IRMM, Retieseweg, Gee l, Belgium) based on the preparation and analysis procedures mentioned in the current work with the reference values of the certified product (Table 2). For the determination of Fe, Zn, Mg, Ca, and K, their R% values were 87.86, 112.09, 106.19, 96.44, and 86.25, respectively; these values are close to 100%. The measured value of Cu was much higher than the certified value; it may have been caused by contamination during sample preparation and handling. The low R% value of Na was possibly due to the volatile loss during the high heat treatment of ICP-AES analysis.

### 3.4. Prediction of Mineral Elements Using PLSR Models

VIP informative variable selection was demonstrated to be the most effective method to select the most relevant Raman spectral variables to enhance the robustness of PLSR models developed for each mineral element prediction. Therefore, results from other informative spectral variable selection algorithms (i.e., sMC and Martens’ uncertainty test) are not discussed in this paper.

#### 3.4.1. PLSR Models Based on All Aqueous INF Samples—Results and Discussion 

**Figure 2:**
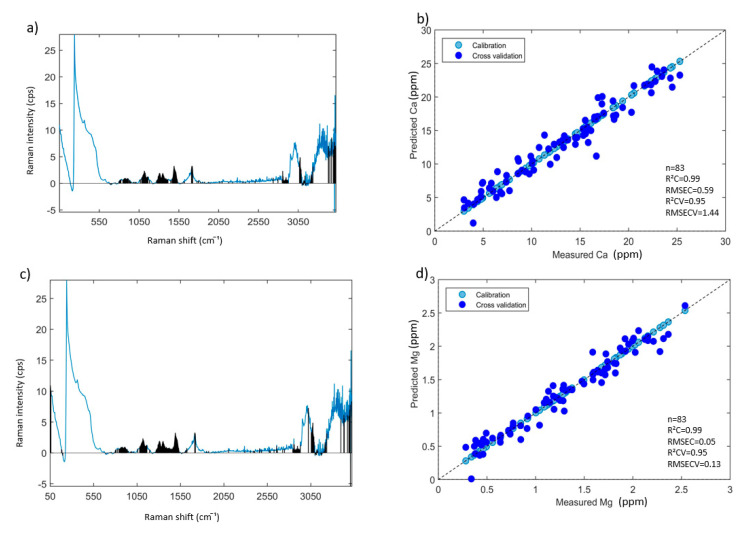

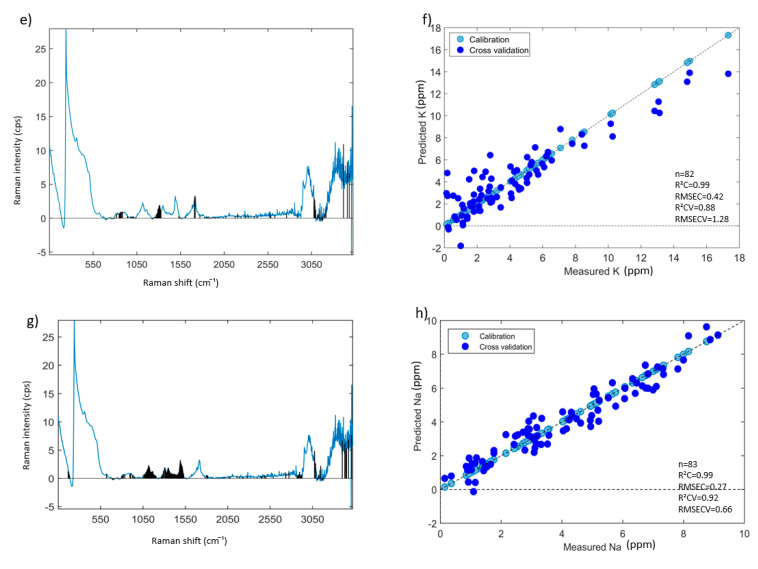

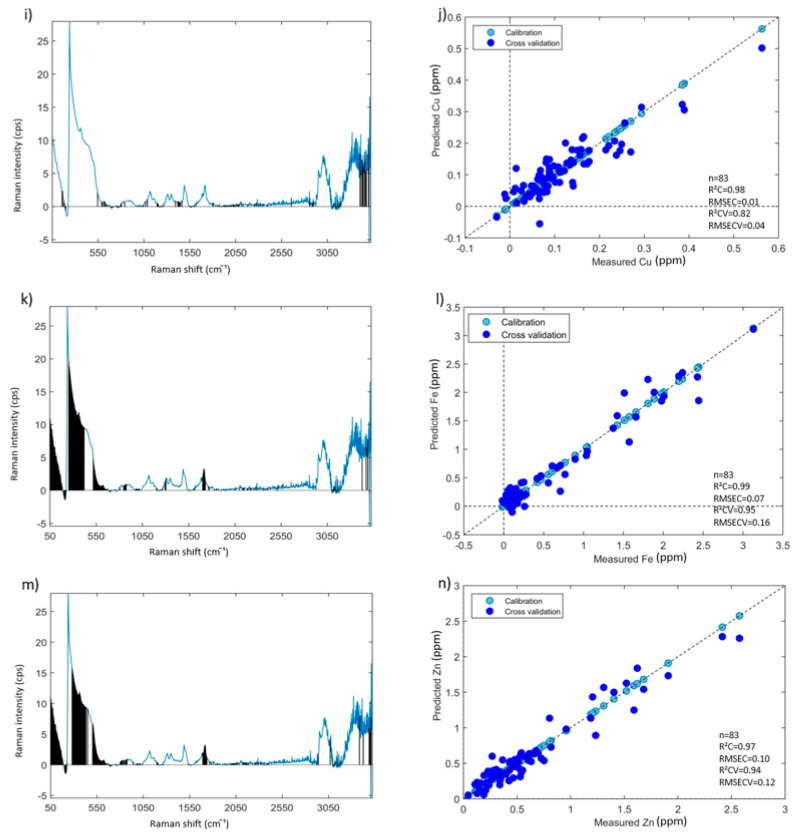
Mean spectrum pre-treated by AsLs baseline correction with spectral variable selection using variable importance on projection (VIP) for the prediction of (**a**) Ca, (**d**) Mg, (**e**) K, (**g**) Na, (**i**) Cu, (**k**) Fe, and (**m**) Zn; PLS regression plots of aqueous INF samples on measured reference values in ppm (X-axis) vs. predicted values in ppm (Y-axis) of (**b**) Ca, (**c**) Mg, (**f**) K, (**h**) Na, (**j**) Cu, (**l**) Fe, and (**n**) Zn. (Note: the spectral variables selected by VIP are shown in black).

Summary results of PLSR models developed with raw Raman spectral data (50–3398 cm^−1^) and data pre-treated respectively using AsLs, air-PLS, BOC, and S.G. first derivatives baseline correction methods are shown in Table 3. Models developed with Raman data pre-treated using AsLs baseline correction performed best for each mineral element prediction. Calibration and cross-validation of PLSR modeling were carried out using all aqueous INF samples (*n* = 83) for the prediction of Ca, Mg, Na, Cu, Fe, Zn. One sample with an unusual ICP-AES measured value of K was eliminated from the PLSR modeling for K prediction. Generally, four or six latent variables were required to attain: R^2^CV values of 0.95 for Ca, Mg and Fe prediction with RMSECV values of 1.44, 0.13 and 0.16 ppm, respectively; an R^2^CV value of 0.94 with an RMSECV value of 0.12 ppm for Zn prediction; R^2^CV values of 0.82, 0.88 and 0.92 with RMSECV values of 0.04, 1.28 and 0.66 ppm for Cu, K, and Na prediction (Figure 2b,d,f,h,j,l,n). 

Minerals and trace elements exist in cows’ milk as ions, salts, or bind with proteins, peptides, carbohydrates, fats, and small molecules to form complexes [49]. In the current study, it was assumed that the mineral binding phenomena in infant formula based on cows’ milk could be observed from the informative variable selection for each mineral prediction. VIP selected Raman spectral variables for the best performing PLSR model of each mineral prediction are shown in Figure 2a,c,e,g,i,k,m. The selected spectral variables for Ca, Mg, K, and Na prediction mainly accumulated around the Raman frequency range of 760–1665 cm^−1^, which are strongly related to the chemical bonds of amino acid structures in proteins and peptides [21,47]. Minerals such as Ca, Mg, K, and Na naturally bind to caseins, lactoferrin, and whey proteins [49]. Raman spectral variables selected for Fe and Zn prediction were mainly observed in the frequency range of 50–600 cm^−1^, which may be related to the broken down O-H bonded structure of water that can be detected near 170 cm^−1^ and possibly hydrolyzed Fe and Zn [50]. Other selected spectral variables around 1654–1665 cm^−1^are related to amide I group of proteins [45]. Fe and Zn also bind to milk proteins in cows’ milk [49]. For the prediction of all eight minerals, the selected spectral variables can be found around 2700–3100 cm^−1^, which is associated with ν (C-H) modes of lipids and >3300 cm^−1^, which is consistent with ν (N-H) and ν (O-H) modes of protein and lactose [20]. 

#### 3.4.2. PLSR Models Developed and Validated Using Calibration and Validation Data Sets—Results and Discussion

To further exploit the potential of RS for mineral content prediction, PLSR models were also developed using two separate calibration and validation sample sets over 50–3398 cm^−1^ and 50–1800 cm^−1^ spectral regions. Models developed based on AsLs baseline-corrected Raman spectra performed best for mineral element prediction. Models developed using VIP selected spectral variables in the Raman frequency range (50–1800 cm^−1^) had similar prediction performance to those developed using spectral variables selected in 50–3398 cm^−1^. A summary of model performances is shown in Table 4. Results reveal R^2^Cs of 0.98, 0.98, 0.99 and 1; R^2^CVs of 0.93, 0.94, 0.97 and 0.94; R^2^Ps of 0.76, 0.77, 0.84 and 0.80 with RMSEPs of 3.05 ppm, 0.29 ppm, 0.30 ppm and 0.25 ppm for Ca, Mg, Fe and Zn prediction, respectively. While for K and Na prediction, R^2^CV values of 0.91 and 0.93 were achieved with RMSECVs of 1.15 ppm and 0.74 ppm and R^2^P values of 0.31 and 0.57 with RMSEPs of 3.45 ppm and 1.51 ppm, respectively. The R^2^P of Cu prediction is 0.08, confirming that the PLSR models failed to predict Cu content in the validation samples. As PLSR models were developed on collinearity of both Raman spectral data (X-variables) and ICP-AES results of each element for each sample (Y- variables), the prediction performances could also reflect the accuracy (R%) of ICP-AES measured values shown in Table 2. R% values of Ca, Mg, Fe, and Zn were closer to 100% (the ideal R% value) than those of Na, K, and Cu. Therefore, the PLSR prediction results of Ca, Mg, Fe, and Zn were also more accurate than those of Na, K, and Cu. Results of ICP-AES analysis may have errors related to volatile elements (e.g., Na and K) loss and ash loss during sample preparation of the dry digestion method. In the current study, both RS and ICP-AES were investigated to determine the trace amount of minerals in aqueous INF samples at low concentrations (<13% *w/w*). RS with chemometrics did demonstrate high sensitivity for the determination of mineral elements. Theoretically, PLSR model prediction performances could be improved using even more accurate chemical reference values (Y- variable) in future studies.

#### 3.4.3. Discussion on Regression Coefficients of PLSR Models for Prediction Mineral Elements

**Figure 3:**
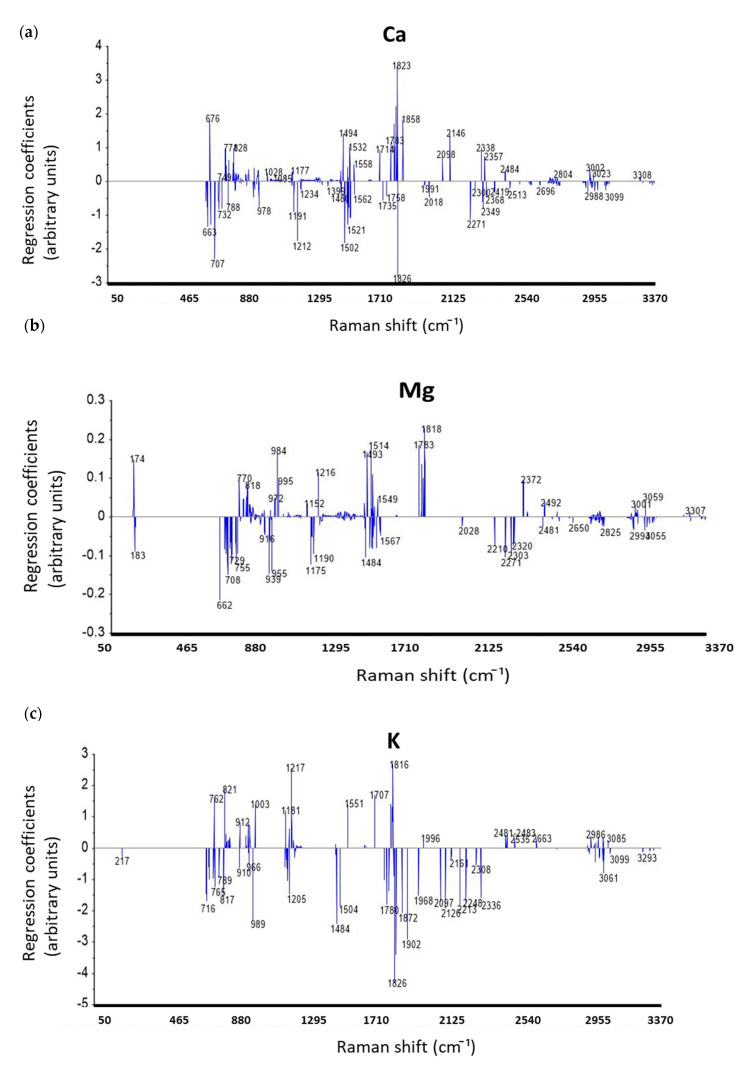

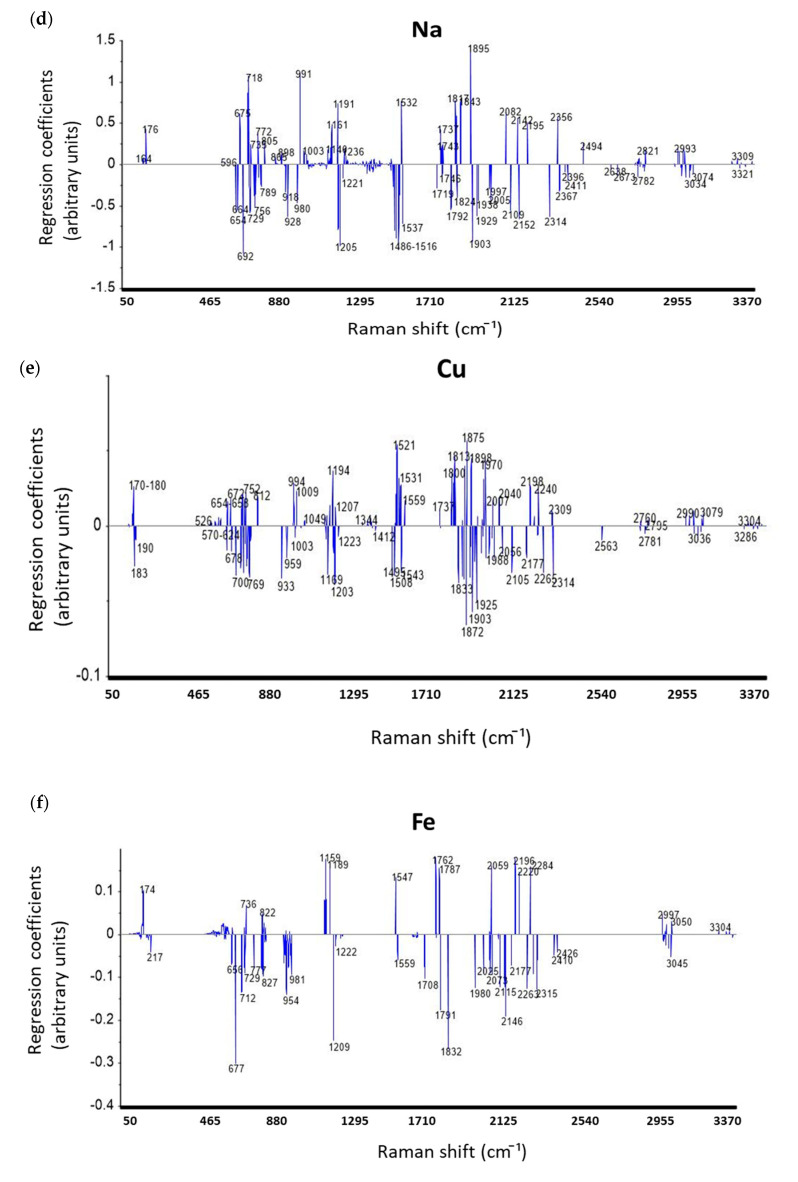

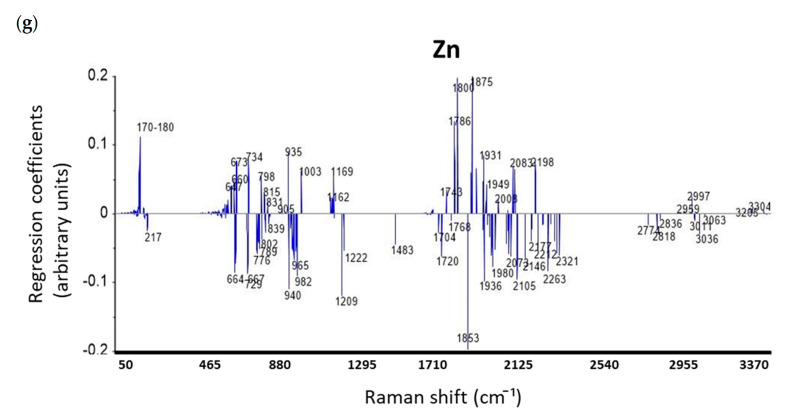
Regression coefficients plots of all aqueous INF samples (*n* = 83) for the prediction of (**a**) Ca, (**b**) Mg, (**c**) K, (**d**) Na, (**e**) Cu, (**f**) Fe, and (**g**) Zn.

The regression coefficient intensity of each wavelength was derived from statistics on the regression results of PLSR modeling. Information on regression coefficients from PLSR modeling is useful for the specification of Raman shifts that related to mineral content. Higher absolute regression coefficient intensities of the wavelengths indicate that these Raman wavelengths are more related to the anions or organic functional groups, which have been bonded with certain mineral ions in studied INF. The intensities of regression coefficients in the spectral range 50–3398 cm^−1^ from PLSR modeling for each mineral element are shown in Figure 3. Generally, all regression coefficient plots show consistently increased intensities from ca. 660 cm^−1^ to ca. 1800–1900 cm^−1^. Patterns of the significant regression coefficient intensities for the prediction of each element are different. However, some significant regression intensities exist at the same Raman shift for different elements’ prediction, e.g., 1003 cm^−1^ assigned to phenylalanine for the prediction of K, Na, Mn, Cu, and Zn. It is assumed that these mineral elements bind to phenylalanine in infant formula samples. Nevertheless, previous publications have rarely mentioned the specific Raman shifts assigned to neutral or ionized minerals. Some previously published studies reported Raman spectral peaks of calcite (CaCO_3_) shown around 709, 1085, 1434, and 1758 cm^−1^ [27,31]; while in the current work, significant regression coefficient intensities also appear at these Raman shifts (Figure 3a). 

**Figure 4:**
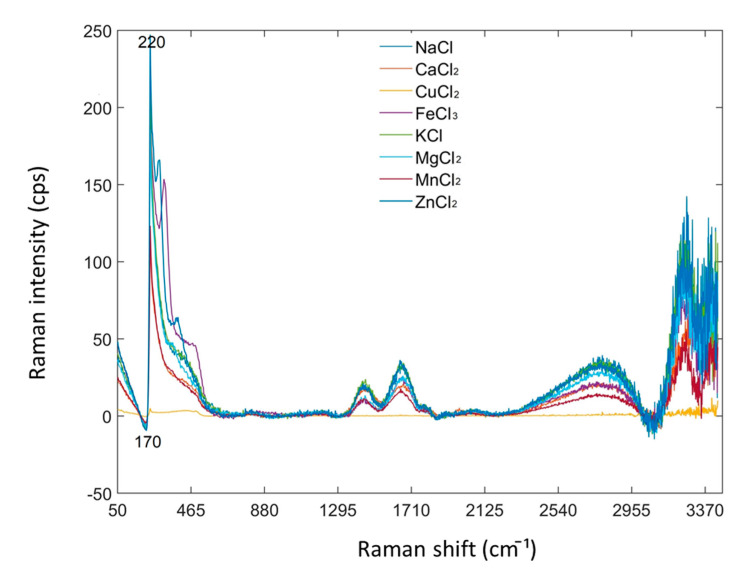
Raman spectra of chloride salt solutions pre-treated by AsLs baseline correction.

Raman spectra of chloride salt solutions (1 M of NaCl, CaCl_2_, CuCl_2_, FeCl_3_, KCl, MgCl_2_, MnCl_2_, and ZnCl_2_) were also collected to observe Raman spectral variations caused by mineral element content. The AsLs baseline-corrected Raman spectra of these chloride salt solutions are illustrated in Figure 4. Differences in these spectra can be observed in the Raman frequency range of 50–3398 cm^−1^, especially in the range of 50–465 cm^−1^. However, the Raman signal noise level was found to increase gradually in the range of 1800–3398 cm^−1^, and the spectral variance of different chloride salt solutions cannot be clearly observed in 1800–3398 cm^−1^. Thus, the Raman signal in this frequency range cannot be used to compare with the corresponding Raman shifts of high regression coefficient intensity in the same frequency range. Correspondingly, significant regression coefficient intensities of the minerals determined (Figure 3b–g) can also be observed at specific Raman shifts around 170–220 cm^−1^ for minerals’ prediction (Figure 4). Therefore, it is aware that 170–220 cm^−1^ of Raman shifts are related to the chelated backbones of Mg, K, Na, Cu, Fe, and Zn ions. These control experiments on chloride salts using RS demonstrated that Raman spectra can be directly used to specify the chelated backbones of multi-minerals. On the other hand, these results also agreed to the method using regression coefficient intensities from PLSR modeling for the identification of the chelated backbones in a chemical complex.

## 4. Conclusions

In this study, the potential of RS with chemometrics was exploited to predict trace amounts of mineral contents in aqueous INF samples. PLSR models developed based on AsLs baseline-corrected Raman spectra in the Raman frequency ranges of 50–1800 cm^−1^ and 50–3398 cm^−1^ have strong potential for mineral content prediction. The reliability of ICP-AES analysis for selected minerals can also be determined by the performance of PLSR modeling based on Raman spectral data and reference values from ICP-AES analysis. Mineral binding effects in dairy-based INF can be identified using VIP informative spectral variable selection algorithm during PLSR modeling. The Raman shifts with significant regression coefficient intensities from PLS regression were demonstrated to be related to the mineral contents determined. 

This study demonstrated the potential of a Raman spectrometer equipped with a fiber optical probe and combined with chemometrics for the determination of multiple minerals in aqueous INF. Consequently, this hypothesis could be implemented to develop a process analytical tool for rapid and real-time determination of mineral content (together with organic components) during the wet mixing process of the INF manufacture. With more engineering innovations the fiber optic probe setting of the Raman spectrometer could allow online monitoring. However, to develop an established process analytical tool for online application, more studies are needed. Future research should investigate a more sophisticated experimental design and modeling using RS and chemometrics for accurate quantification of multiple mineral elements in INF.

## Figures and Tables

**Table 1:** Mineral elements expressed as ppm in aqueous INF samples (*n* = 83) determined using ICP-AES.

Elements	ICP-AES Wavelength (nm)	Min (ppm)	Max (ppm)	Mean (ppm)	Median (ppm)	Repeatability (ppm)
Cu	324.754	0.009	0.563	0.117	0.088	0.003
Fe	238.204	0.016	3.13	0.512	0.142	0.013
Mn	257.61	−0.109	0.487	0	−0.01	0.001
Zn	213.657	0.045	2.575	0.573	0.426	0.009
Mg	279.553	0.281	2.537	1.275	1.28	0.034
Ca	422.673	3.002	25.295	13.413	13.522	0.032
Na	588.995	0.144	9.133	4.055	3.571	0.034
K	766.491	0.129	17.319	4.227	2.919	0.017

Min—minimum; Max—maximum.

**Table 2:** Comparison of ICP-AES measured values and certified skim milk powder (ERM-BD 150).

Element	Certified Value ^1^	Measured Value ^2^	Mean Recovery (%)
Cu (mg/kg)	1.08 ± 0.06	1.94 ± 0.29	181.69
Fe (mg/kg)	4.6 ± 0.5	3.91 ± 0.76	87.86
Mn(mg/kg)	0.289 ± 0.018	−0.56 ± 0.04	−206.64
Zn (mg/kg)	44.8 ± 2.0	49.90 ± 4.82	112.09
Mg (g/kg)	1.26 ± 0.1	1.32 ± 0.12	106.19
Ca (g/kg)	13.9 ± 0.8	13.32 ± 0.71	96.44
Na (g/kg)	4.18 ± 0.19	1.87 ± 0.49	45.37
K (g/kg)	17 ± 0.7	14.62 ± 0.40	86.25

^1^ Mean ± S.D., S.D., standard deviation; ^2^ Mean ± S.D., S.D., standard deviation.

**Table 3:** Summary of venetian blinds cross-validation PLSR performances (Raman frequency: 50–3398 cm^−1^) for mineral elements prediction. (most accurate models in bold).

	Data Type	Raman Frequency (cm^−1^)	Calibration Samples	Spectral Variables	# PLS Loadings	R^2^C	RMSEC	R^2^CV	RMSECV	Bias
Ca	**AsLs**	**50–3398**	**83**	**618**	**6**	**0.99**	**0.59**	**0.95**	**1.44**	**0**
	air-PLS	50–3398	83	605	4	0.92	1.77	0.86	2.36	0.074
	BOC	50–3398	83	782	4	0.86	2.37	0.84	2.53	0.041
	S.G. 1st der. 7sm	50–3398	83	708	5	0.96	1.2	0.87	2.29	0.033
	Raw	50–3398	83	1856	5	0.91	1.93	0.86	2.39	0.031
Mg	**AsLs**	**50–3398**	**83**	**683**	**6**	**0.99**	**0.05**	**0.95**	**0.13**	**−0.002**
	air-PLS	50–3398	83	725	5	0.93	0.15	0.88	0.21	0.002
	BOC	50–3398	83	2611	4	0.86	0.23	0.83	0.25	−0.002
	S.G. 1st der. 7sm	50–3398	83	707	3	0.86	0.23	0.82	0.26	0.007
	Raw	50–3398	83	736	4	0.89	0.2	0.87	0.22	0.005
K	**AsLs**	**50–3398**	**82**	**195**	**6**	**0.99**	**0.42**	**0.88**	**1.28**	**−0.029**
	air-PLS	50–3398	82	1329	3	0.56	2.44	0.44	2.78	0.027
	BOC	50–3398	82	3473	4	0.55	2.46	0.41	2.86	0.021
	S.G. 1st der. 7sm	50–3398	82	525	2	0.54	2.48	0.47	2.7	0.001
	Raw	50–3398	82	311	4	0.47	2.67	0.36	2.96	0.027
Na	**AsLs**	**50–3398**	**83**	**463**	**6**	**0.99**	**0.27**	**0.92**	**0.66**	**−0.038**
	air-PLS	50–3398	83	302	3	0.71	1.26	0.66	1.36	0.012
	BOC	50–3398	83	2611	4	0.87	0.22	0.85	0.24	0
	S.G. 1st der. 7sm	50–3398	83	622	2	0.74	1.18	0.71	1.27	0.012
	Raw	50–3398	83	794	4	0.76	1.14	0.67	1.36	0.003
Cu	**AsLs**	**50–3398**	**83**	**262**	**6**	**0.98**	**0.013**	**0.82**	**0.04**	**−0.001**
	air-PLS	50–3398	83	1433	5	0.75	0.05	0.31	0.08	0
	BOC	50–3398	83	2681	2	0.38	0.07	0.34	0.08	0
	S.G. 1st der. 7sm	50–3398	83	778	3	0.61	0.06	0.29	0.08	0.002
	Raw	50–3398	83	3356	2	0.38	0.07	0.33	0.08	0
Fe	**AsLs**	**50–3398**	**83**	**643**	**5**	**0.99**	**0.07**	**0.95**	**0.16**	**0.003**
	air-PLS	50–3398	83	410	3	0.91	0.22	0.89	0.24	−0.001
	BOC	50–3398	83	3473	4	0.9	0.22	0.87	0.26	0.001
	S.G. 1st der. 7sm	50–3398	83	372	4	0.95	0.16	0.91	0.22	0.006
	Raw	50–3398	83	3356	4	0.9	0.22	0.87	0.26	0.003
Zn	**AsLs**	**50–3398**	**83**	**683**	**4**	**0.97**	**0.1**	**0.94**	**0.12**	**−0.001**
	air-PLS	50–3398	83	569	3	0.83	0.21	0.78	0.24	0.003
	BOC	50–3398	83	715	5	0.99	0.05	0.94	0.12	−0.009
	S.G. 1st der. 7sm	50–3398	83	686	5	0.92	0.14	0.87	0.18	0.003
	Raw	50–3398	83	192	2	0.76	0.25	0.7	0.28	−0.005

PLSR, partial least square regression; AsLs, asymmetric least squares correction; airPLS, adaptive iteratively reweighted penalized least squares; BOC, baseline off correction; S.G., Savitzky–Golay; der., derivatives; sm, smoothing points; #, number of PLS loading; R^2^C, coefficient determination of calibrations; RMSEC, root mean square error of calibration; R^2^CV, correlation coefficient of determination in venetian blinds cross-validation; RMSECV, root mean square error of venetian blinds cross-validation.

**Table 4:** Summary of venetian blinds cross-validation PLSR performances (Raman frequency: 50–3398 cm^−1^ and 50–1800 cm^−1^) based on AsLs baseline corrected Raman spectra for mineral elements prediction (most accurate models in bold).

	Raman Frequency (cm^−1^)	Spectral Variables	Calibration Samples	#PLS Loadings	R^2^C	RMSEC	Bias	R^2^CV	RMSECV	Bias	Validation Samples	R^2^P	RMSEP	Bias
Ca	50–3398	910	42	5	0.99	0.62	0.000	0.93	1.75	−0.186	41	0.76	3.05	−0.456
	**50–1800**	**322**	**42**	**4**	**0.98**	**0.96**	**0.000**	**0.93**	**1.68**	**−0.017**	**41**	**0.75**	**3.17**	**−0.205**
Mg	50–3398	450	42	3	0.98	0.10	0.000	0.95	0.15	0.006	41	0.75	0.29	−0.027
	**50–1800**	**476**	**42**	**4**	**0.98**	**0.08**	**0.000**	**0.94**	**0.15**	**−0.007**	**41**	**0.77**	**0.29**	**−0.035**
K	50–3398	87	41	5	0.95	0.87	0.000	0.75	1.95	0.025	41	0.22	3.80	0.800
	**50–1800**	**125**	**41**	**7**	**0.99**	**0.30**	**0.000**	**0.91**	**1.15**	**−0.099**	**41**	**0.31**	**3.45**	**1.120**
Na	50–3398	317	42	4	0.99	0.24	0.000	0.93	0.63	0.005	41	0.57	1.54	0.241
	**50–1800**	**211**	**42**	**6**	**0.99**	**0.20**	**0.000**	**0.90**	**0.74**	**0.014**	**41**	**0.6**	**1.51**	**0.235**
Cu	50–3398	620	42	5	0.98	0.01	0.000	0.75	0.04	−0.029	41	0.08	0.11	−0.029
	**50–1800**	**181**	**42**	**6**	**0.99**	**0.01**	**0.000**	**0.85**	**0.03**	**−0.001**	**41**	**0.04**	**0.11**	**−0.025**
Fe	**50–3398**	**466**	**42**	**4**	**0.99**	**0.07**	**0.000**	**0.97**	**0.12**	**0.004**	**41**	**0.84**	**0.30**	**0.008**
	50–1800	211	42	6	0.99	0.20	0.000	0.90	0.74	0.014	41	0.6	1.51	0.235
Zn	**50–3398**	**1465**	**42**	**5**	**0.99**	**0.03**	**0.000**	**0.94**	**0.12**	**0.000**	**41**	**0.8**	**0.25**	**0.019**
	50–1800	869	42	6	1.00	0.03	0.000	0.93	0.12	−0.006	41	0.76	0.26	0.014

PLSR, partial least square regression; AsLs, asymmetric least squares correction; #, number of PLS loading; R^2^C, coefficient determination of calibrations; RMSEC, root mean square error of calibration; R^2^CV, correlation coefficient of determination in venetian blinds cross-validation; RMSECV, root mean square error of venetian blinds cross-validation; R^2^P, correlation coefficient of determination in prediction; RMSEP, root mean square error of prediction.
